# The Evolutionary Trend of Global Inequality: Analyzing the Impacts of Economic Structure

**DOI:** 10.3389/fpsyg.2021.808976

**Published:** 2021-12-09

**Authors:** Ning Ma, Victor Jing Li, Tsun Se Cheong, Delin Zhuang

**Affiliations:** ^1^School of Financial Management, Hainan College of Economics and Business, Haikou, China; ^2^Department of Geography and Resource Management, The Chinese University of Hong Kong, Sha Tin, Hong Kong SAR, China; ^3^Department of Economics and Finance, Hang Seng University of Hong Kong, Sha Tin, Hong Kong SAR, China; ^4^School of Economics, Hefei University of Technology, Hefei, China

**Keywords:** inequality, global, decomposition analysis, economic structure, Gini coefficient

## Abstract

The aim of this study is to examine the evolution of inequality by focusing on the impacts of the economic structure. The technique of decomposition by income sources is employed to evaluate the contribution of the three major sectors, namely the agricultural, industrial, and service sectors to overall inequality. The data cover almost all the countries in the world from 2001 to 2017 for a total of 18 years. There are four stages of analysis in this study. The first stage of study is to provide an overall view of the evolutionary trend of global inequality, the second stage focuses on the North-South divide, the third stage determines the impacts of income groups, and the fourth stage investigates the impacts for each region. There are several salient findings: First, global inequality had declined in the study period. Second, the service sector is identified as the largest contributor to global inequality, followed by the industrial sector, while the contribution of the agricultural sector is negligible. For the North-South divide, disparity in the service sector was more marked in the North than in the South. The industrial sector played a major role in the South and contributed more than 40% to overall inequality. For the comparison amongst the income groups, our findings show that the higher the income, the higher the percentage contribution of the service sector (except for the low-income group). Finally, for the comparison across regions, although the contribution of the agricultural sector in most regions are below 1.5%; however, the contribution of the agricultural sector in both Sub-Saharan Africa and South Asia is more than 8%. It implies that a lot of people in these regions still rely on the agricultural sector for a living, and the development in the industrial and service sectors in these two regions lagged behind those of the other regions. Our analysis show that the evolution pattern is very different for each region, therefore, it is necessary to take the effects of income and geographical location into consideration in formulating development policies.

## Introduction

The General Assembly of the UN adopted the resolution for a new set of Sustainable Development Goals on 25 September 2015. Including 17 Sustainable Development Goals (SDGs) and 169 targets, the 2030 Agenda is unprecedentedly ambitious, universal and overarching. At the core of the Agenda is the determination to eradicate poverty and hunger in all their forms, which is within the reach of this generation for the first time in human history (Hong, 2016). The changes in UN’s agenda call for a detailed research on the thorny issue of inequality so that policy implications can be drawn to assist countries in formulating inequality-alleviating policies. As global inequality was integrated into the new agenda, it marks the start of a new era following the conclusion of the Millennium Development Goals (MDGs). This calls for a comprehensive research on this area so that policy implications can be drawn to assist the attempt of mitigating international income inequality.

The aim of this study is to examine the evolutionary trend of international income inequality. Given that inequality can exert various adverse impacts on society and regional stability, the United Nations (UN) integrated the issue of inequality into the new set of Sustainable Development Goals (SDGs) in 2015. The new agenda formulated by the UN provides an outline of the roadmap of human development up to 2030, and the UN expects that the new goals and targets will stimulate global action in the years ahead. This study aims to provide relevant information and policy suggestions concerning international income inequality so as to contribute to the literature in the post-MDGs era by employing decomposition analysis and studying the underlying transitional dynamics behind the trend.

This paper is divided into two research components. First, Gini indices are computed for the world to provide an overview of the evolutionary patterns and trends of international inequality. Second, the technique of decomposition by income sources is employed to evaluate the contributions of the three economic sectors. The information on the relative significances of the agricultural, industrial and service sectors can facilitate the formulation of industrial policy for economic development in the developing countries. The decomposition analysis not only can shed light on the underlying patterns of inequality, but also reveal the relationship between geographical groupings and inequality, thereby pinpointing the crux of the problem of inequality in full detail.

The remainder of this paper is organized as follows. Section 2 provides a literature review on international income inequality. Section 3 offers relevant information on methodology and the data employed in this study. Results and discussions are provided in Section 4, while Section 5 summarizes the research findings with policy implications.

## Literature Review

Inequality has been identified as the root cause of social and political instability in many countries (Muller and Seligson, 1987; Alesina and Perotti, 1996; Wang and Hu, 1999; Acemoglu and Robinson, 2001; Wen, 2007; Dutt and Mitra, 2008; Wilkinson and Pickett, 2009; Knight, 2013). In a report prepared by the World Economic Forum (2012), the researcher sternly warned that, “… when ambitious and industrious young people start to feel that, no matter how hard they work, their prospects are constrained, then feelings of powerlessness, disconnectedness and disengagement can take root. The social unrest that occurred in 2011, from the United States to the Middle East, demonstrated how governments everywhere need to address the causes of discontent before it becomes a violent, destabilizing force.” Actually, not only the United States and the Middle East countries were affected; on the other side of the globe, many western countries also suffer from similar issues as the levels of inequality have increased substantially. Many countries are troubled by inequality, and public discontent with the governments in these countries remained at a high level. It is not surprising that the researcher claimed that the rising inequality was one of the top “global risks” of the world (World Economic Forum, 2012).

Income inequality is relatively stable within countries but varies significantly among countries (Li et al., 1998; Goesling, 2001; Frazer, 2006; Acemoglu and Dell, 2010). Regarding the level of income inequality, Anand and Segal (2008) reviewed literature in the 1990s and concluded that the Gini coefficient was between 0.63 and 0.69 at that time. It is worth noting that “the concentration of world income in the wealthiest quintile (fifth) of the world’s population is indeed shocking and cannot meet any plausible test of legitimacy” (Wade, 2001). However, global inequality tends to decline due to the economic rise of China and south Asian countries’ industrialization (Firebaugh and Goesling, 2004; Ferreira and Ravallion, 2008; Hung and Kucinskas, 2011).

Specifically, from 1820s to 1950s there was a co-movement of per capita income and increasing global inequality, which remained more stable afterward with a unimodal distribution in the nineteenth century and between 1980 and 2000; but the pattern had become more bimodal between 1910s and 1970s (Van Zanden et al., 2014). Korzeniewicz and Moran (1997) maintained that cross country inequality is the most significant component of overall world income inequality from 1965 to 1992. Similarly, Sala-i-Martin (2002) found a reduction in the global income inequality from 1980 to 1998, and most of the global disparities can be attributed to across country rather than within country inequalities (Firebaugh, 2000). However, this declining trend may be diverted again if developing countries do not start growing (Sala-i-Martin, 2002; Jensen and Rosas, 2007; Meschi and Vivarelli, 2009; Gasparini et al., 2011; Fosu, 2017). Milanovic (2012) found similar declining trend of global income inequality, however, he claimed that the main driving factor to sustain the trend is mean income convergence which can reduce the huge citizenship premium of the rich countries. Lakner and Milanovic (2013) found that the global Gini Coefficient had dropped by two points from 1988 to 2008. Rougoor and Van Marrewijk (2015) projected that global income inequality will enlarge due to the differences in population growth and population structure across countries. The differences in the decomposition of global income inequality may be due to how cross country income comparisons are made (Dowrick and Akmal, 2005; Fosu, 2010; Ortiz and Cummins, 2011); or the quality of the database (Deininger and Squire, 1996; Chotikapanich et al., 1997; Atkinson and Brandolini, 2001; Babones and Alvarez-Rivadulla, 2007; Solt, 2009, 2016; Jenkins, 2015; Alvaredo et al., 2017). Other related issues in the time of pandemic are policy uncertainty and globalization. Fang et al. (2021) found that policy uncertainty is correlated with economic globalization negatively. Given that globalization is an important driving factor behind the economic structure, policy uncertainty may also affect inequality. Chen et al. (2021) suggested that the World Pandemic Discussion Index (WPDI) is positively associated with income inequality in 34 OECD economies but negatively associated with income inequality in non-OECD economies, thereby suggesting that global inequality may deteriorate further with the pandemic, thereby calling for an investigation into the evolution of global inequality.

Many researchers maintain that inequality exerts an adverse effect on poverty reduction (Wade, 2004; Rupasingha and Goetz, 2007; Zhuang, 2008; Fosu, 2009; Corak, 2013); while other researchers claim that inequality has a negative impact on economic growth and financial stability (Chase-Dunn, 1975; Alesina and Rodrik, 1994; Persson and Tabellini, 1994; Alesina and Perotti, 1996; Deininger and Squire, 1998; Mahler, 2004; Huang et al., 2009; Helpman et al., 2010; Lysandrou, 2011; Van Treeck and Sturn, 2012). Cheong and Wu (2015) reported that there is a positive correlation between inequality and the crime rate. Likewise, other researchers found that inequality can lead to various types of social dysfunction, including social unrest, mental illness, racism, and even political upheaval (Muller and Seligson, 1987; London and Robinson, 1989; Alesina and Perotti, 1996; Wang and Hu, 1999; Acemoglu and Robinson, 2001; Kentor, 2001; Mahler, 2004; Wen, 2007; Dutt and Mitra, 2008; Wilkinson and Pickett, 2009; Knight, 2013). Given that income inequality has so many adverse impacts, a comprehensive study on inequality is justified so that policies can be formulated to alleviate inequality and ameliorate these adverse effects. However, to our knowledge, no recent study has been conducted on the contributions of income sources to overall global income inequality. Therefore, this study aims to fill the gap in the literature by investigating the trend of global inequality through a series of decomposition so as to reveal the relationship between economic structure and global income inequality.

## Methodology and Data

### Inequality Measurement

A lot of inequality measurements are available; however, the most common ones are the Gini coefficient because it satisfies the property of the Pigou-Dalton condition and income-zero-homogeneity (Bourguignon, 1979). The Pigou–Dalton principle suggests that a transfer of income from the rich to the poor should result in a fall of the inequality indicator, so long as the transfer does not reverse the ranking of the rich and the poor in the income distribution (Cheong, 2012). Income-zero-homogeneity means that the value of the inequality measurement should remain constant for a scale change of the whole income distribution (Cheong, 2012).

The Gini coefficient is based on the Lorenz curve, which plots the cumulative share of income against the cumulative share of population from the lowest to highest incomes. The Gini coefficient is the ratio of the area that lies between the uniform distribution line and the Lorenz curve over the total area under the uniform distribution line. The value of the Gini coefficient ranges from zero to one. The value of one corresponds to perfect income inequality, whilst the value of zero corresponds to perfect income equality.

The formulae of population-weighted Gini coefficient is


(1) $$\sum_{i}\sum_{j}\frac{\left|y_{i}-y_{j}\right|}{2\mu }\frac{n_{i}}{N}\frac{n_{j}}{N}$$

where *N* is total population in the world, *n*_*i*_ and *n*_*j*_ are population in country *i* and country *j*, respectively, *y*_*i*_ and *y*_*j*_ are GDP per capita in country *i* and country *j*, respectively, and


$$\mu =\sum_{i}\frac{y_{i}n_{i}}{N}\left(\mathrm{Tsui, 1996}\right)$$

The formula of the unweighted Gini coefficient is


(2) $$\sum_{i}\sum_{j}\frac{\left|Y_{i}-Y_{j}\right|}{2\mu R^{2}}$$

where *R* is total number of countries, *Y*_*i*_ and *Y*_*j*_ are GDP in country *i* and country *j*, respectively, and $\mu =\sum_{i}\frac{Y_{i}}{R}$.

### Decomposition by Income Sources

The approach of decomposition by income sources is employed in this study as it has many advantages. First, this approach can decompose Gini coefficient completely, and Gini coefficient is deemed to be the most common inequality measurement used in the literature, thereby facilitating comparisons amongst different studies. Second, the contributions of all the three major industries can be derived, therefore, it can highlight the importance of each industry and unveil the relationship between inequality and economic structure. Third, by conducting decomposition by income sources across time, the trend and evolution of inequality due to changes in economic structure can be examined in full detail.

A country’s GDP is made up of the value added generated from the three economic sectors. The Gini coefficient can be decomposed into these income sources so as to calculate the contribution of each sector to overall international inequality.


(3) $$G=\sum_{s=1}^{S}W_{s}Cs$$

where *G* is international income inequality as measured by the Gini coefficient, *C*_*s*_ is the concentration coefficients of sector *s*, and *W*_*s*_ is the income share of sector *s* in total income.

So *G* is the weighted sum of concentration coefficients which can be computed by:


(4) $$C_{s}=1-\sum_{j=1}^{m}P_{j}\left(2Q_{sj}-W_{sj}\right)$$

where $Q_{sj}=\sum_{l=1}^{j}W_{sl}$, *W*_*sj*_ is the share of income for country *j* in total source income *s*, and *P*_*j*_ is the population share in total population for country *j*. Both *P*_*j*_ and *W*_*sj*_ should be sorted in ascending order of total income per capita in the calculation of *C*_*s*_. The contribution of each sector can be calculated as:


(5) $$\mathrm{Contribution}\mathrm{of}\mathrm{sector}s=\frac{W_{s}C_{s}}{G}$$

### Data

The output value of the three sectors, namely, the agricultural, industrial and service sectors, as well as the data of GDP and population were compiled from the World Development Indicator Database of the World Bank. It is notable that it is necessary to employ the same countries for calculating the Gini coefficient for every year because the addition or omission of a country in a particular year may distort the relative importance of other countries in that year. Therefore, the countries employed in this study are the same for every year in the study period. Except the value of GDP, the output value generated by each sector should also be employed in the computation of the relative contribution to global inequality, therefore, a few countries are not included in this study because of the output value of the three sectors are not available. However, almost all the countries listed in the World Bank World Development Indicators Database are included in this study. It is worth noting that it is important to maintain the same set of countries in the study period to ensure that the changes in inequality are due to the changes in GDP per capita rather than a change in the composition of the countries. Since some data are missing in later years, the study period spans from 2001 to 2017 for 17 years.

This study is divided into several stages: First, the Gini coefficient was calculated for the full dataset so as to offer an overhead view of the evolution of inequality from 2001 to 2017. Then the data were divided into two smaller datasets, namely, the North and South groups so as to examine the North-South divide. In the third stage of analysis, the dataset were separated into four income groups. The grouping method is based on the four income groups as defined by the World Bank, namely, low, lower-middle, upper-middle, and high income groups. Finally, the full dataset were divided into six regional groups according to the regional classification proposed by the World Bank, namely, the East Asia and Pacific, Europe and Central Asia, Latin America and Caribbean, Middle East and North Africa, South Asia, and Sub-Saharan Africa. This classification allows one to evaluate the impact of industrial policy on inequality within these regional groups. However, it is worth noting that the North America region is excluded from this study because decomposition cannot be performed satisfactorily since the number of countries in this region is too few. In fact, there are only three countries located in North America, namely, Canada, the United States, and Mexico.

## Results and Discussion

### Global Inequality

**FIGURE 1:**
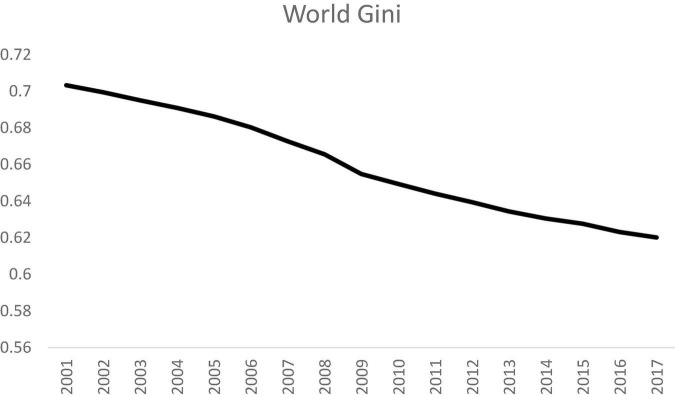
Gini coefficient for the world, 2001 to 2017. Source: Authors’ calculation.

Figure 1 shows the evolutionary trend of the Gini coefficient for all the countries in the world from 2001 to 2017. It can be observed that the Gini coefficient had dropped from 0.70 to 0.62 for an 11.8% change in this study period. This is an encouraging finding as it implies that the disparity amongst the countries had narrowed steadily across time. This is a good beginning for the implementation of the SDGs, and it sets the stage for future international cooperation in mitigating inequality.

**FIGURE 2:**
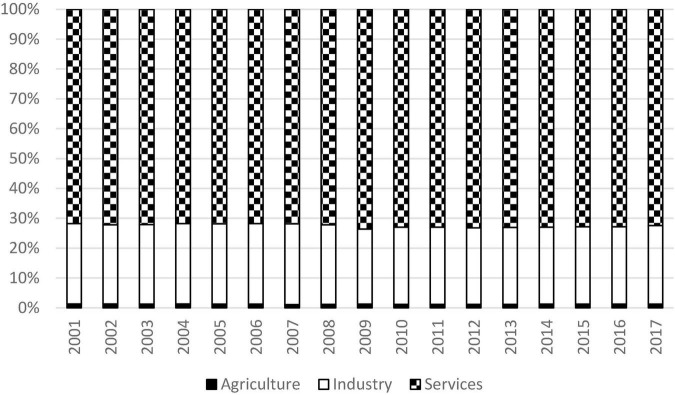
Decomposition of inequality for the world, 2001 to 2017. Source: Authors’ calculation.

Decomposition by income sources was then performed to evaluate the contribution of each sector to overall inequality. The results are shown in Figure 2. The percentage contribution of both the agricultural and industrial sectors had dropped slightly, and the service sector had increased a little. Surprisingly, it shows that the percentage contribution of each sector is fairly constant across time. 72.4% of global inequality can be explained by the disparity within the service sector, 26.4% can be explained by the inequality within the industrial sector, while the agricultural sector only contributed 1.2% to overall inequality in 2017.

This is a very important finding and it suggests that the uneven distribution of output of the service sector is the crux of the problem of inequality in the world. Industrial sector also contributed a lot to overall inequality, however, the magnitude is much less and it is only one third of that of the service sector. Combining together, the total contribution of the two sectors is more than 98%, while the contribution of the agricultural sector is negligible. This finding pinpoints the importance of the industrial and service sectors. The governments in the developing countries should divert more resources to these two sectors and encourage further investments in them so as to promote long-term economic growth.

### North-South Divide

**FIGURE 3:**
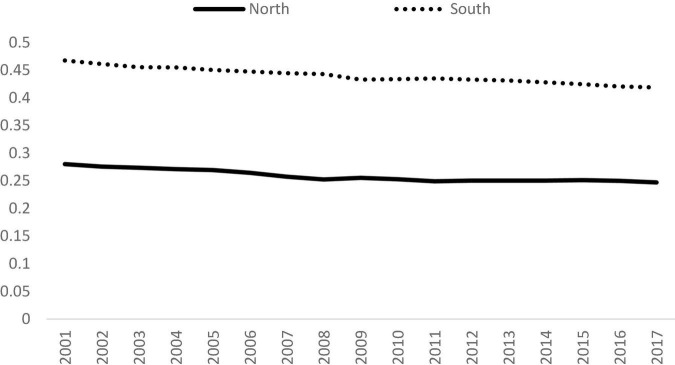
Gini coefficients for the North and South, 2001 to 2017. Source: Authors’ calculation.

The next stage of this study is to examine the North-South Divide. The trends of the Gini coefficient of the North and South are shown in Figure 3. It can be observed that the inequality within the South was higher than that of the North. However, the inequalities in both regions declined in the study period. The Gini coefficient of the South fell from 0.47 in 2001 to 0.42 in 2017, while the Gini coefficient of the North dropped from 0.28 to 0.25. It shows that the disparity within both regions had narrowed across time. However, the large disparity within the developing countries is a warning sign for pursuing the SDGs.

**FIGURE 4:**
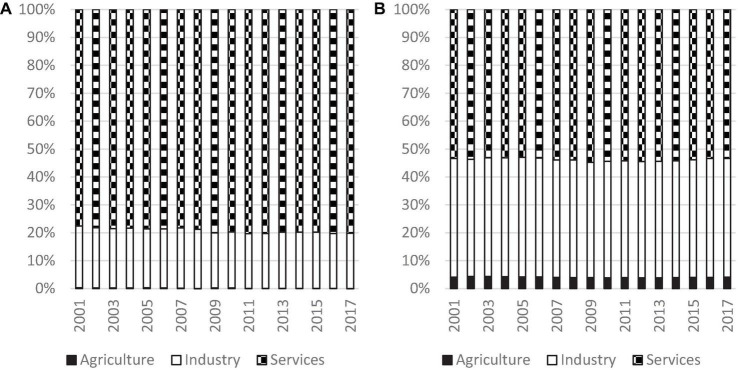
Decomposition of inequality for the North and South, 2001 to 2017. Source: Authors’ calculation. **(A)** North and **(B)** South.

Apart from the evolutionary trend of inequality, the next burning issue is whether the classification of North and South has any impacts on the contribution of the three sectors to overall inequality. Figure 4 shows the decomposition results for each group in detail. It can be observed that in 2017, the largest contributor for the North was the service sector and its contribution was more than 80%, while industrial sector was about 20% and contribution of the agricultural sector is negligible. Moreover, the contribution of the service sector had increased from 77.6% in 2001 to 80.2% in 2017, and the contribution of the other two sectors dropped slightly in that period, thereby suggesting that the service sector had gained in relative importance in terms of overall inequality. This is a rather distributing finding as it suggests that the situation may deteriorate further for the developed countries which are already subject to the high disparity in output of the service sector.

Turning to the South, the largest contributor in 2017 was also the service sector, and its contribution to overall inequality was 53.4%. The contribution of the industrial sector was 42.5% and agricultural sector was 4.1% in that year. Although service sector contributed the most, however, the industrial sector also played a major role in inequality. The two sectors contributed more than 95%. It is notable that the contribution of the three sectors in the South remained fairly constant in the study period.

In summary, by comparing Figures 4A,B, it can be concluded that the service sector was the largest contributor to overall inequality for both groups, followed by the industrial sector, while the contribution of the agricultural sector was negligible. The huge disparity in the service sector was more marked in the North than in the South. Moreover, the findings show that the situation had worsened across time. It is also worth mentioning that the industrial sector played a major role in the South and contributed more than 40% to overall inequality, thereby highlighting the fact that the governments of developing countries should also focus on industrial development rather than focusing only on the service sector.

### Comparison Across Income Groups

**FIGURE 5:**
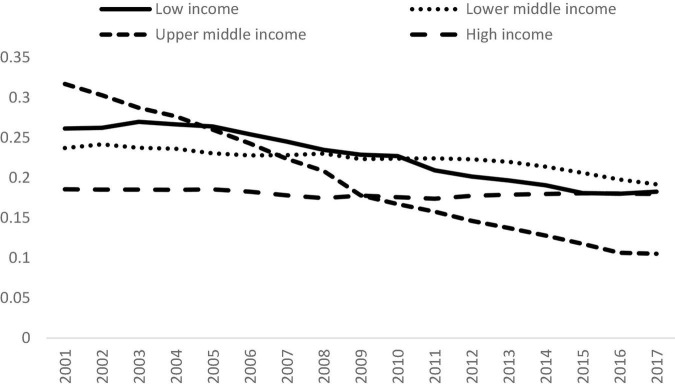
Gini coefficients for different income groups, 2001 to 2017. Source: Authors’ calculation.

The data were separated into four datasets based on the income level of a country according to the scheme proposed by the World Bank. The countries were grouped into four income groups, namely, the low-income, the lower-middle, the upper-middle, and the high income groups. The evolution of the trend is shown in Figure 5. Back in 2001, the upper-middle-income group had the highest level of inequality amongst the four, and its Gini coefficient was 0.32, followed by the low-income group with a Gini coefficient of 0.26. The Gini coefficient in the lower-middle-income group was 0.24, and the high-income group had the lowest level of inequality (0.19) in 2017.

Surprisingly, the inequalities within all the income groups had declined. The Gini coefficient for the low-income countries fell from 0.26 in 2001 to 0.18 in 2017 with a reduction of more than 30%. This is an encouraging finding and it shows that the disparity amongst the poor countries was reduced considerably. Turning to the lower-middle-income countries, the Gini coefficient dropped from 0.24 to 0.19 with a fall of 19%. The decline was moderate compared with that of the low-income group.

Figure 5 shows that there was a huge drop in the inequality within the upper-middle-income group. The Gini coefficient dropped from 0.32 to 0.11 in the study period, with an overall reduction of 67%. This is an extraordinary achievement and the countries within this group had a significant decline in overall inequality across time. The upper-middle-income countries had the lowest level of inequality amongst the four income groups due to this improvement in 2017. This finding shows that international inequality can be mitigated, thereby suggesting that the SDGs are within reach and can be achieved through international cooperation and careful planning of development policy.

Turning to the high-income countries, although the Gini coefficient had dropped from 0.19 to 0.18, the decline is only 3.2%. In fact, the reduction in inequality within the high-income countries was the smallest amongst the four income groups. It shows that the progress in inequality alleviation was much slower than the other income groups.

**FIGURE 6:**
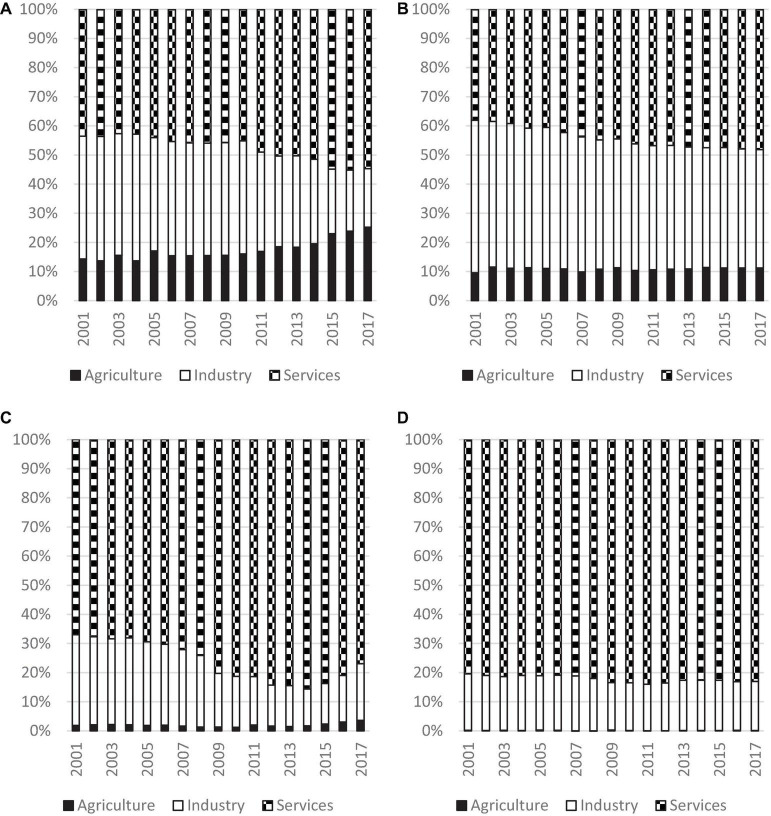
Decomposition of Inequality for Different Income Groups, 2001 to 2017. Source: Authors’ calculation. **(A)** Low, **(B)** Lower-Middle, **(C)** Upper-Middle, and **(D)** High.

The decomposition results of the four income groups are shown in Figure 6. It can be observed that the change in contribution is very different for all the income groups. For the low-income subgroup, the percentage contribution of the agricultural sector increased from 14.3 to 25.2% resulting in an increase of 10.9%, the contribution of the service sector increased from 43.5 to 54.7% for an increase of 11.2%, while the industrial sector suffered from a huge decline. The contribution of the industrial sector fell from 42.2 to 20.1% resulting in a decline of 22.1%. This is unanticipated as it shows that the role of the industrial sector had been reduced in the study period. Almost half of the decline in contribution of the industrial sector was replaced by the increase of the contribution of the service sector, however, it is surprising to note that the other half was due to the rise in contribution of the agricultural sector.

Figures 6B,C shows the decomposition results of the lower-middle-income and the upper-middle-income groups, respectively. They exhibited similar patterns to the low-income group. The contribution of the industrial sector had declined in the study period, while the contribution of the agricultural and service sectors had increased. It can be observed that the increase in contribution of the service sector was higher than the agricultural sector for all the three income groups.

Turning to the high-income group, Figure 6D shows that the contribution of the service sector had decreased within the study period. However, the contribution of both agricultural and industrial sectors fell within that period. In fact, only the agricultural sector in the high-income group showed a drop in contribution, while the agricultural sector in all the other three income groups showed an increase. However, the drop in contribution of the agricultural sector is negligible for the high-income group as the contribution was 0.23% in 2001 and 0.13% in 2017, resulting in only a decline of 0.1%.

It is of interest to compare the evolution patterns of all the income groups. There are some interesting observations: First, the largest contributor to inequality was identified to be the service sector for all the income groups. Second, for the high-income, upper-middle-income, and lower-middle-income groups, the second highest contributor was the industrial sector in 2017, while the agricultural sector contributed the smallest amount. However, it is a little different for the low-income group as the contribution of the agricultural sector was higher than that of the industrial sector. Third, except for the low-income group, it can be observed that the higher the income, the higher the percentage contribution of the service sector. The contribution of the service sector was 83.0% for the high-income group, 76.9% for the upper-middle-income group, 48.2% for the lower-middle-income group and 54.7% for the low-income group. Fourth, the contribution of the service sector had increased, while the contribution of the industrial sector had declined for all the income groups. Finally, the contribution of the agricultural sector had declined for the high-income group, while the contribution of this sector had increased for all other income groups, thereby signifying the insignificance of the agricultural sector for the high-income group.

### Comparison Across Regions

**FIGURE 7:**
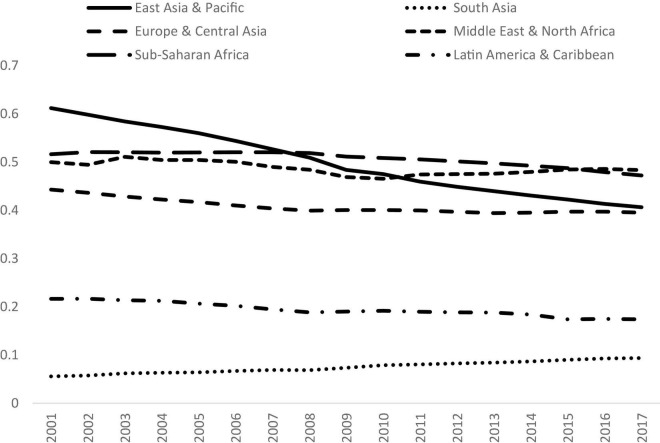
Gini coefficients for different regions, 2001 to 2017. Source: Authors’ calculation.

The next stage of this study is to examine the impacts of regional groupings on the contribution of inequality. The results are provided in Figure 7. It is notable that the inequalities in almost all the regions had declined in the study period, however, the inequality in South Asia had risen. In 2017, Middle East and North Africa had the highest level of inequality (0.48), followed by Sub-Saharan Africa (0.47). The inequality in East Asia and Pacific (0.41) was similar to that in Europe and Central Asia (0.40). Latin America and Caribbean had the second lowest inequality (0.17), while South Asia had the lowest level of inequality within its region (0.09). It shows that the characteristics are very different for the regions, thereby calling for further investigation for each region individually.

**FIGURE 8:**
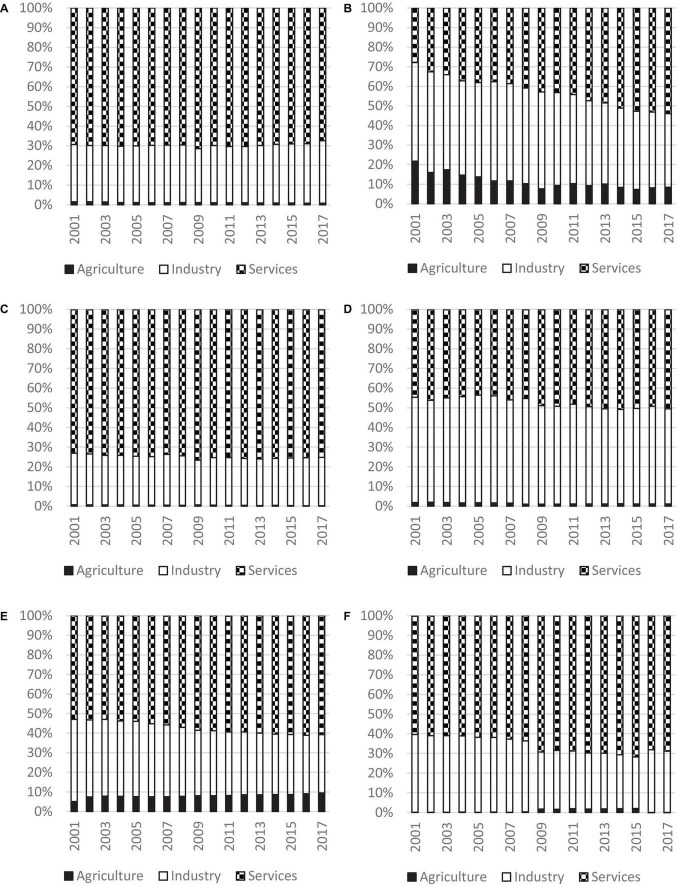
Decomposition of inequality for different regions, 2001 to 2017. Source: Authors’ calculation. **(A)** East Asia and Pacific, **(B)** South Asia, **(C)** Europe and Central Asia, **(D)** Middle East and North Africa, **(E)** Sub-Saharan Africa, and **(F)** Latin America and Caribbean.

Decomposition by income sources were conducted for each region individually, and the results are shown in Figure 8. It can be observed that the contribution of the service sector had increased for all the regions except that of the East Asia and Pacific. It is notable that East Asia and Pacific is quite unusual as the contribution of the service sector had declined from 2001 to 2017, while that of the industrial sector had increased. In fact, East Asia and Pacific is also very special in terms of industrial development. The contribution of the industrial sector had increased for East Asia and Pacific, but decreased for all the other regions. Turning to the agricultural sector, the contribution of that sector had increased for Sub-Saharan Africa, but decreased for all other regions.

There are several other salient findings. First, the largest contributor to inequality was the service sector for all the regions. The contribution was 75.2% for Europe and Central Asia which had the highest level of contribution in 2017, while Middle East and North Africa had the lowest level of contribution and the value was 50.6%. It suggests that service sector had played a major role in global income inequality and contributed more than 50% to overall inequality for all the regions. Second, the industrial sector is identified to be the second largest contributor to overall inequality in all the regions, thereby signifying the importance of the industrial sector. Third, the agricultural sector contributed the least amount to overall inequality. The contribution of the agricultural sector in most regions are below 1.5%; however, it was 9.5% in Sub-Saharan Africa, and 8.4% in South Asia. It implies that a lot of people in these regions still rely on the agricultural sector for a living, and the development in the industrial and service sectors in these two regions lagged behind those of the other regions.

In summary, although the service is deemed to be the most important player in regional inequality, East Asia and Pacific is getting more and more susceptible to the disparity in industrial development, while Sub-Saharan Africa is prone to the inequality within the agricultural sector. These findings suggest that each of the region has very different pattern of evolution of inequality, therefore, it is necessary to take geographical location into consideration in formulating development policies.

## Conclusion

It is worth noting that the data used in this study does not cover the period after the spread of COVID-19. Given that global inequality can be aggravated further as many developing countries may suffer more from the pandemic due to a lack of resources, one can expect that global inequality may be much worse than the results derived from this study in the short run. However, in the long run, it can be expected that inequality should be driven by the economic structure when things resume back to normal after the pandemic.

The scale and ambition of SDGs “requires a revitalized Global Partnership to ensure its implementation. This Partnership will work in a spirit of global solidarity. It will facilitate an intensive global engagement in support of implementation of all the Goals and targets, bringing together Governments, the private sector, civil society, the United Nations system and other actors and mobilizing all available resources” (Hong, 2016). In response to this general principle, this paper explores several crucial issues so as to enrich the knowledge base of inequality by employing decomposition analysis and studying the underlying transitional dynamics behind the trend.

This study aims to study the evolution of inequality by focusing on the impacts of the economic structure. The technique of decomposition by income sources is employed to evaluate the contribution of the three major sectors, namely the agricultural, industrial, and service sectors to overall inequality. The data were compiled from the World Bank. The data cover almost all the countries in the world from 2001 to 2017 for a total of 18 years. There are four stages of analysis in this study. The first stage of study is to provide an overall view of the evolutionary trend of global inequality, the second stage focuses on the North-South divide, the third stage determines the impacts of income groups, and the fourth stage investigates the impacts for each region. This approach enables us to study the contribution of the three sectors in great detail, so that pertinent policy implications can be derived from the results.

There are several salient findings: First, global inequality had declined in the study period. Second, the service sector is identified as the largest contributor to global inequality, followed by the industrial sector, while the contribution of the agricultural sector is negligible. For the North-South divide, disparity in the service sector was more marked in the North than in the South. The industrial sector played a major role in the South and contributed more than 40% to overall inequality, thereby suggesting that the governments of developing countries should also focus on industrial development.

For the comparison amongst the income groups, our findings show that the higher the income, the higher the percentage contribution of the service sector (except for the low-income group). The contribution of the service sector had increased, while the contribution of the industrial sector had declined for all the income groups. Interestingly, the contribution of the agricultural sector had declined for the high-income group, while the contribution of this sector had increased for all other income groups.

Finally, for the comparison across regions, although the contribution of the agricultural sector in most regions are below 1.5%; however, the contribution of the agricultural sector in both Sub-Saharan Africa and South Asia is more than 8%. It implies that a lot of people in these regions still rely on the agricultural sector for a living, and the development in the industrial and service sectors in these two regions lagged behind those of the other regions. Our analysis show that the evolution pattern is very different for each region, therefore, it is necessary to take the effects of income and geographical location into consideration in formulating development policies. This study not only can reveal the evolution of inequality and the contribution of each sector across time, but also can provide relevant information for allocating scarce resources in promoting economic development.

## Data Availability Statement

The original contributions presented in the study are included in the article/supplementary material, further inquiries can be directed to the corresponding author/s.

## Author Contributions

NM: data collection, data preparation, and writing. VL: literature review, data cleaning, and writing. TC: conceptualization, data curation, methodology, and software. DZ: discussion, writing, and editing. All authors contributed to the article and approved the submitted version.

## Conflict of Interest

The authors declare that the research was conducted in the absence of any commercial or financial relationships that could be construed as a potential conflict of interest.

## Publisher’s Note

All claims expressed in this article are solely those of the authors and do not necessarily represent those of their affiliated organizations, or those of the publisher, the editors and the reviewers. Any product that may be evaluated in this article, or claim that may be made by its manufacturer, is not guaranteed or endorsed by the publisher.
